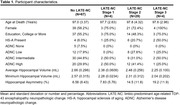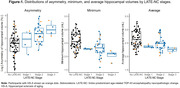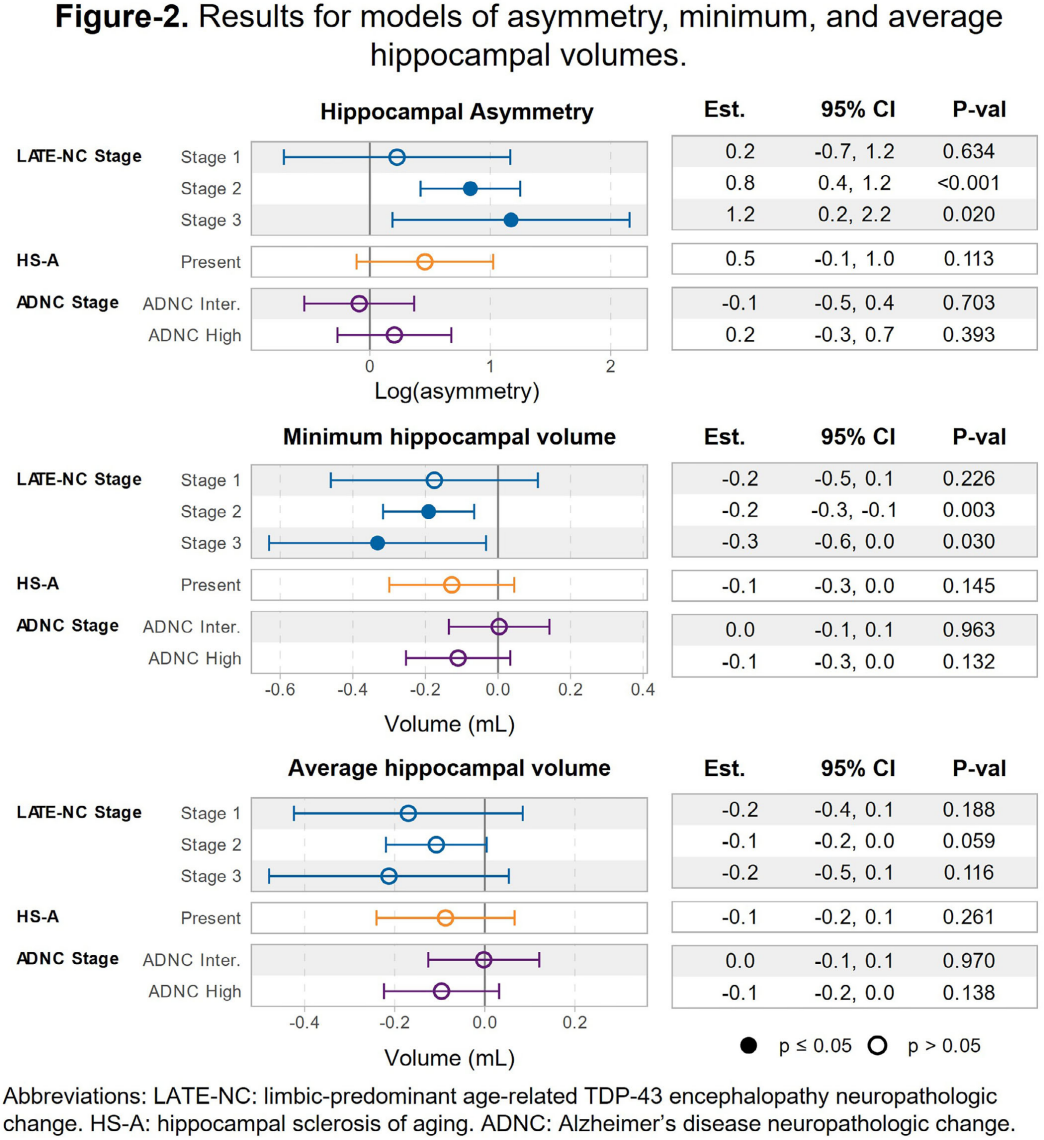# Asymmetrical hippocampal atrophy: an arrow in the quiver of imaging markers for LATE‐NC

**DOI:** 10.1002/alz.090878

**Published:** 2025-01-03

**Authors:** Davis C. Woodworth, Dana E. Greenia, Syed A. Bukhari, Thomas J. Montine, Ali Ezzati, Claudia H. Kawas, María M. M. Corrada, S. Ahmad Sajjadi

**Affiliations:** ^1^ University of California, Irvine, Irvine, CA USA; ^2^ Stanford University, Stanford, CA USA

## Abstract

**Background:**

Limbic‐predominant age‐related TDP‐43 encephalopathy neuropathologic change (LATE‐NC) denotes TDP‐43 deposition in older age and is consequential for cognitive function. Currently there is no way to identify LATE‐NC during life. Some forms of TDP‐43 deposition in younger age, related to frontotemporal dementia (FTD), are associated with pronounced asymmetrical atrophy of the temporal lobe. Given the similar underlying proteinopathy of TDP‐43 in both LATE‐NC and FTD, we hypothesized LATE‐NC would be associated with asymmetrical hippocampal atrophy.

**Methods:**

We included participants from *The 90+ Study* with both MRI and autopsy data. All participants were assessed for LATE‐NC, Alzheimer’s disease neuropathologic change (ADNC), and hippocampal sclerosis of aging (HS‐A). 3D‐T1w MRIs were segmented using FreeSurfer. We examined hippocampal asymmetry, and minimum and average hippocampal volumes (across left and right), in relation to LATE‐NC stages. We defined asymmetry as *asym* = log(100*abs(left. hipp. vol.‐right. hipp.vol)/(ave. hipp. vol)). We fit multiple linear regression models for each outcome, accounting for HS‐A presence and ADNC severity. Lastly, we examined prediction performance using area under the curve (AUC) for LATE‐NC stage 2 or higher, considering dementia status alone or including asymmetry, minimum, or average hippocampal volume. All models were adjusted for age at death, sex, education, and intracranial volume.

**Results:**

**Table‐1** displays participant characteristics (N = 104), with N = 37 (36%) having LATE‐NC. **I**ncreasing LATE‐NC stage was associated with increasing hippocampal asymmetry and decreasing minimum and average hippocampal volumes (**Figure‐1**). LATE‐NC stages 2 and 3 showed significant associations with hippocampal asymmetry (**Figure‐2**). HS‐A trended towards an association with, but ADNC was not associated with, hippocampal asymmetry (**Figure‐2**). Both asymmetry and minimum hippocampal volume appeared to be more strongly related to LATE‐NC compared to average hippocampal volume (**Figure‐2**). AUC analysis indicated that, for predicting LATE‐NC stage 2 or greater, the model which included asymmetry (AUC = 0.79) outperformed both the model with dementia status alone (AUC = 0.64, P = 0.005) and the models with average (AUC = 0.65, P = 0.007) and minimum (AUC = 0.69, P = 0.014) hippocampal volumes.

**Conclusion:**

LATE‐NC is associated with asymmetrical hippocampal atrophy in a stage‐dependent fashion, independent of HS‐A and ADNC. Using measures of hippocampal asymmetry may lead to better identification of LATE‐NC during life.